# Appropriate Models for the Management of Infectious Diseases

**DOI:** 10.1371/journal.pmed.0020174

**Published:** 2005-07-26

**Authors:** Helen J Wearing, Pejman Rohani, Matt J Keeling

**Affiliations:** **1**Institute of Ecology, University of Georgia, Athens, Georgia, United States of America,; **2**Department of Biological Sciences, University of Warwick, Coventry, United Kingdom; Cornell UniversityUnited States of America

## Abstract

**Background:**

Mathematical models have become invaluable management tools for epidemiologists, both shedding light on the mechanisms underlying observed dynamics as well as making quantitative predictions on the effectiveness of different control measures. Here, we explain how substantial biases are introduced by two important, yet largely ignored, assumptions at the core of the vast majority of such models.

**Methods and Findings:**

First, we use analytical methods to show that (i) ignoring the latent period or (ii) making the common assumption of exponentially distributed latent and infectious periods (when including the latent period) always results in underestimating the basic reproductive ratio of an infection from outbreak data. We then proceed to illustrate these points by fitting epidemic models to data from an influenza outbreak. Finally, we document how such unrealistic a priori assumptions concerning model structure give rise to systematically overoptimistic predictions on the outcome of potential management options.

**Conclusion:**

This work aims to highlight that, when developing models for public health use, we need to pay careful attention to the intrinsic assumptions embedded within classical frameworks.

## Abstract 2

Two current biases in infectious disease models may substantially affect their usefulness in predicting disease oubreaks.

## Introduction

The past decade has seen a dramatic increase in the significance attached to infectious diseases from the public health perspective. This trend is due in part to the emergence of new and highly pathogenic infections such as Ebola [1], West Nile virus [2], and SARS [3]. There are also well-publicized concerns surrounding the deliberate introduction of pathogens as bioterrorism weapons [4,5], and the continued persistence and resurgence of older infections, several of which now boast strains resistant to more than one drug [6]. In addition, there have been a number of high-profile and economically expensive disease outbreaks in domestic livestock [7–9] as well as wildlife populations [10].

The effective management and control of such infections is increasingly done with substantial input from mathematical models, which are used not only to provide information on the nature of the infection itself, through estimates of key parameters such as the basic reproductive ratio *R*_0_ [11], but also to make predictions about the likely outcome of alternative courses of action [12–15]. During the 2001 outbreak of foot-and-mouth disease in the United Kingdom, for example, the former UK Ministry of Agriculture, Fisheries, and Food set up a committee that included two groups with expertise in mathematical modeling of disease dynamics. It is becoming increasingly important, therefore, that epidemiological models produce accurate quantitative predictions, and this in turn relies on accurate parameterization. Here, we examine the dynamical consequences of an unrealistic yet almost ubiquitous assumption embedded in such models concerning the distribution of the latent and infectious periods. In particular, we show that without greater care in model formulation, we may risk systematic biases when parameterizing models from data and may make overly optimistic policy recommendations.

**Figure 1:**
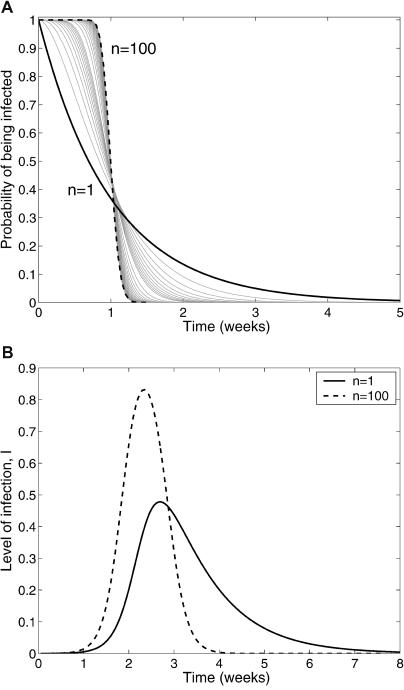
Gamma-Distributed Infectious Periods and Their Effects on the Epidemic Curve (A) The change in the probability of remaining infectious as a function of time when the number of subdivisions within the infected class increases from *n =* 1 to *n =* 100. Irrespective of the value of *n,* the mean duration of the infectious period is 1 wk. When *n =* 1, the distribution of the infectious period is exponential, but as *n* increases the infectious period becomes closer to a constant length. (B) The consequences of changes in *n* for the SIR-type epidemic without births or deaths. For the same basic reproductive ratio, *R*_0_ = 5, and the same average infectious period, *γ =* 1, larger values of *n* lead to a steeper increase in prevalence and an epidemic of shorter duration.

The most commonly used framework for epidemiological systems, is still the susceptible–infectious–recovered (SIR) class of models, in which the host population is categorized according to infection status as either susceptible, infectious, or recovered [16,17]. Subsequent refinements of the model have incorporated an additional exposed (infected but not yet infectious) class (susceptible–exposed–infectious–recovered [SEIR] models) (see Protocol S1 for mathematical equations). One of the fundamental mathematical assumptions in such models is that the rate of leaving the exposed or infectious class is constant, irrespective of the period already spent in that class. While mathematically very convenient, this assumption gives rise to exponentially distributed latent and infectious periods, which is epidemiologically unrealistic for most infections [18–20]. A more sensible formulation would be to specify the probability of leaving a class as a function of the time spent within the class, such that initially the chance of leaving the class is small, but the probability increases as the mean infectious/latent period is reached. This would give rise to a more realistic distribution of latent and infectious periods, with a stronger central tendency. A convenient way to describe such distributions is to write an expression for the infectious class (neglecting the latent class for this example) as follows:



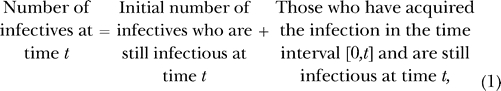



which translates mathematically into



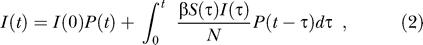



where *β* is the infection transmission rate and *N* is the total population size. The probability of remaining infectious through time is governed by the survivorship function, *P*(*t*), and as such the average infectious period, denoted by 1/*γ,* is given by 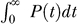 [21]. The probability density function of the infectious period, *p*(*t*), is just the negative derivative of the survivorship function, −*dP*(*t*)/*dt*. Different functional forms for *p*(*t*) give rise to alternative assumptions concerning the distribution of the infectious period in the model. For example, setting *p*(*t*) equal to e^−*γ*t^/*γ* recovers the classical exponentially distributed SIR model. More realistic distributions can be obtained by choosing *p*(*t*) to be a gamma probability density function [22–27], with parameters *γ* and *n* (see Protocol S1). An alternative (and computationally efficient) means of modeling infections with gamma distributions is to divide the infectious class into *n* subclasses with *nγ* being the rate of sequential progression through the subclasses. The advantage of this formalism is that when *n* = 1 we recover the exponentially distributed model, which has a large variance, while as *n→∞* we obtain a fixed infectious period. The effects of *n* on the distribution of the infectious period are demonstrated in Figure 1A; in Table 1 we present some examples of latent and infectious period distributions estimated from data.

**Table 1:**
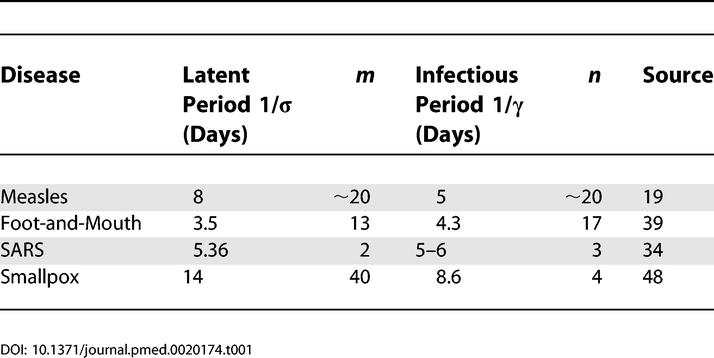
Estimates of Latent and Infectious Period Durations Estimated from Data for a Number of Infections, Together with the Associated Gamma-Distribution Parameter (*m* and *n*)

The dynamical consequences of these differences in the distribution of infectious and latent periods have received some attention over the past two decades. It has been shown, for example, that the precise distribution of the infectious period has no qualitative effects on the asymptotic values or properties of the system [21,24], though perturbations to the endemic equilibrium take longer to die out as *n* increases [22,26]. When contact rates vary seasonally, for example, to mimic the aggregation of children in schools [28,29], changes in *p*(*t*) are known to have important consequences for the persistence likelihood of infections [25,26,30]. An issue that has received surprisingly little attention, despite its obvious applicability to emerging infections and possible “deliberate exposure,” is the influence of latent and infectious period distributions on the invasion dynamics of an infection into a largely susceptible population. This is in contrast to the conceptually similar situation of within-host dynamics of viral disease, such as HIV, for which some models already adopt realistic distributions to describe stages in the cell life cycle [31,32]. In particular, Lloyd [33] has shown how parameter estimates made from viral load data are affected by different assumptions about these distributions. Here we are interested in the application of this work to between-host transmission dynamics. As can be seen in Figure 1B, changes in the gamma distribution parameter *n* have substantial quantitative consequences for the epidemic curve: in comparison to a gamma-distributed model, the epidemic given by the exponentially distributed model (i) takes off at a dramatically slower rate, (ii) predicts a significantly smaller (approximately 56%) peak number of cases, and (iii) lasts much longer (almost twice as long).

Whether these marked differences between alternate model formulations may translate into potentially important public health concerns is a key question, which we address in two ways. First, we document systematic differences in the model parameters estimated from an epidemic using the exponential and gamma-distributed models. Second, we demonstrate that the use of exponential models produces overoptimistic predictions about the low levels of control required to subdue an epidemic.

## Methods

### The Relationship between *R*_0_ and Initial Epidemic Growth Rate

During the early phase of an epidemic, the observed exponential growth rate, *λ,* is related to the basic reproductive ratio, *R*_0_, of the infection. Mathematically, *λ* is just the dominant eigenvalue of the disease-free equilibrium, and one can show that *λ* must satisfy



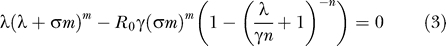



for the SEIR model with gamma-distributed latent and infectious periods (further details are given in Protocol S1). This equation translates into an expression for *R*_0_ in terms of *λ* and the other parameters, as is presented in equation 4. Therefore, if we can estimate the growth rate *λ* from data, we can use equation 4 to obtain an estimate of *R*_0_.

### Contact Tracing and Isolation

To study the effects of contact tracing and isolation, we modify the assumptions of the SEIR epidemic model, while still incorporating gamma-distributed latent and infectious periods. In the new model, isolation of newly infectious cases occurs at a daily rate of *d_I_* after a delay of *τ_D_* days, which represents a period when infected individuals are infectious but asymptomatic or undetectable *(I_A_)*. A fraction *q* of those who had contact with an infectious and symptomatic individual *(I_S_)* (but did not contract the infection) are removed to the quarantined susceptible class, *S_Q_,* where they spend exactly *τ_Q_* days. An identical fraction of newly exposed individuals is also quarantined. Full details of the model equations are given in Protocol S1.

## Results

In a typical management setting, such as the SARS outbreak of 2003, public health professionals are confronted with a novel (or perhaps a highly virulent variant of an existing) pathogen that is spreading rapidly through a predominantly susceptible population. One of the important tasks of any modeling exercise is to provide insights into some of the epidemiological characteristics of the invading infection, such as its transmissibility, virulence, and persistence dynamics. Of great interest is the estimation of the basic reproductive ratio of the infection (*R*_0_), which measures the transmission potential of the infection, and determines the degree of control required.

Some of these aspects can be explored by studying the range of model parameters that give (initial) outbreak dynamics consistent with the (short-term) epidemic data thus far gathered. One approach is to fit model parameters to data by “trajectory matching,” where one looks for the combination of parameters that, in a statistically rigorous sense, give rise to dynamics most consistent with observed patterns [34,35]. Alternatively, one may use the well-established result that during the initial stages of an epidemic, the growth rate is approximately exponential [17,20], with the rate determined by *R*_0_. First, we use this approach to examine, in general, how the distribution of the latent and infectious periods may affect the estimation of *R*_0_ from initial epidemic data. To illustrate that our results are not specific to this methodology, we then take incidence data from an influenza outbreak and parameterize the epidemic models using trajectory matching.

### Estimating *R*_0_ from Initial Epidemic Growth Rate

We may obtain an estimate for *R*_0_ by calculating the initial growth rate *(λ)* of an infection from data and equating it to the growth rate of the equations, calculated from the dominant eigenvalue of the disease-free equilibrium (see Methods). Such an exercise reveals that for any observed *λ,* the precise value of *R*_0_ estimated is crucially dependent on the fundamental assumptions made concerning the distributions of latent and infectious periods. Specifically, we find that the following equation determines the relationship between *R*_0_ and an empirically estimated epidemic growth rate, *λ:*



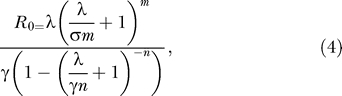



where *m* and *n* represent the number of subclasses in the exposed and infectious categories, respectively. The mean latent and infectious periods are represented by 1/*σ* and 1/*γ,* respectively, and are assumed to be known or estimated from independent data. This relationship was first determined by Anderson and Watson [36] and has recently been applied in the context of viral life cycle dynamics by Lloyd [33].

**Figure 2:**
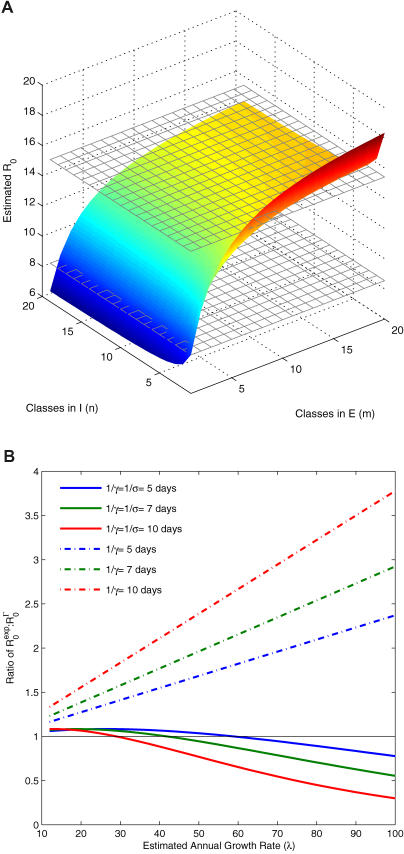
Estimates of *R*_0_ from Data on the Initial Growth Rate of an Epidemic (A) The effects of changing the distributions of the latent and infectious periods on the estimated value of *R*_0_, with *λ* assumed to be 100 per year and the average latent and infectious periods fixed at 1 wk. The gray grid surfaces show the asymptotic values of *R*_0_ when the latent and infectious periods are both exponentially distributed (lower surface) or fixed (higher surface). We note that the shape of each surface is independent of the exact value of *λ*. (B) At higher values of λ, *R*_0_ may be substantially over- or underestimated using the classical exponentially distributed model (*n = m =* 1) compared to periods of fixed lengths (*n = m→∞*), depending on whether an exposed class is included (solid lines) or not (dashed lines).

The relationship between estimated *R*_0_ and the distributions of the latent and infectious periods is demonstrated in Figure 2A. It reveals a subtle yet very important interaction between model structure and estimated *R*_0_. In general, as the infectious period becomes more tightly distributed (increasing *n*), lower values of *R*_0_ are estimated for any given growth rate *λ*. On the other hand, as the variance in the latent period is reduced (increasing *m*), *higher* values of *R*_0_ are estimated. Indeed, we may use the relationship given by equation 4 to arrive at the following general principle: if we ignore the latent period, then models with an exponentially distributed infectious period will always overestimate the infection's basic reproductive ratio. When the latent period is included, however, this finding is reversed when the growth rate is large (Figure 2B). In closely examining equation 4, we note that the basic reproductive ratio estimated from a model without an exposed class (1/*σ* = 0) is always lower than the estimate from the corresponding model when a latent period is included (1/*σ* > 0) (see equation S1 in Protocol S1). Therefore, when faced with a rapidly spreading infection, either entirely ignoring the latent period or assuming exponential distributions will lead to an underestimate of *R*_0_ and therefore will underestimate the level of global control measures (such as mass vaccination) that will be needed to control the epidemic.

### Estimating *R*_0_ from Trajectory Matching

**Figure 3:**
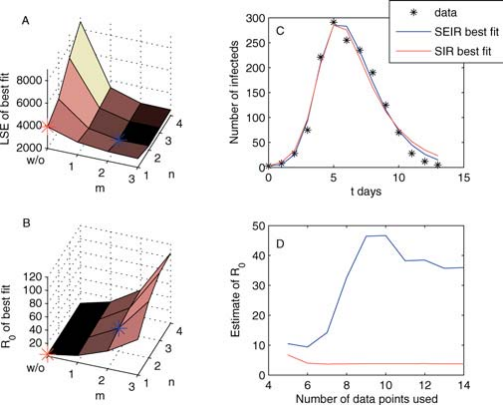
Fitting Epidemic Models to Data from an Influenza Outbreak (A and B) The least squares error (LSE) (A) and *R*_0_ (B) of the best-fit model under different assumptions about the distribution of the latent and infectious periods. (The label “w/o” denotes no latent class.) (C) We plot the incidence data along with the SEIR best fit (*m =* 2, *n =* 2) and that obtained by ignoring any latent period (*n =* 1)—the SIR best fit. (D) The best-fit estimate of *R*_0_ changes for these two models as we increase the number of points used in the fitting procedure. When fitting the models, for each value of *n* (and *m*), we are estimating the average infectious period, 1/*γ,* and transmission parameter, *β* (and average latent period, 1/*σ*). The effective population size for the influenza outbreak was known to be *N =* 763.

While the results described in the previous section are based on the rate of epidemic take-off, we reach the same qualitative conclusions about the effects of the distributions of latent and infectious periods when estimating *R*_0_ by other data-fitting methods. For illustration, we use data from an influenza outbreak in an English boarding school [37] to estimate model parameters by trajectory matching. In the absence of independent data, this method can be used to provide estimates of the key infectious parameters. Of course, here we can also compare the parameter estimates to observed parameter ranges, since the influenza virus is known to have a latent period of between 1 and 4 d and infected individuals may transmit the virus up to 4 or 5 d after the onset of illness [38]. We determine the best fit of the model output to daily incidence data by minimizing the least squares errors for different values of the distribution parameters *m* and *n*. For comparison, we also determine the best-fit parameters in the absence of any latent period. The least squares errors and estimated *R*_0_ of the best fit for a combination of *m* and *n* are presented in Figure 3. These results clearly illustrate the points raised in the previous section: (i) entirely ignoring the latent period gives a significantly lower estimate of *R*_0_, and (ii) the assumption of exponentially distributed latent and infectious periods results in consistently lower estimates of *R*_0_ than their gamma-distributed counterparts.

Despite visually similar solutions, the SIR best-fit and SEIR best-fit models (Figure 3C) result in strikingly different estimates of *R*_0_: 3.74 for the SIR model versus 35.9 for the SEIR model, which is partly a result of the small population size. However, the best-fit estimate of *R*_0_ obtained from the gamma-distributed SEIR model (*m* = 2, *n* = 2) is much more sensitive to the number of points used to obtain the fit (Figure 3D): the exponentially distributed SIR model gives the same estimate whether the first six (up to the peak in incidence) or more points are used. This difference is further emphasized if we use the first few points of the data to estimate the rate of epidemic take-off (*λ* = 1.0837 d^−1^), and then take the final estimates of the average latent and infectious periods to compute *R*_0_ using equation 4. For the SIR model (*n =* 1, 1/*γ* = 2.2 d) we obtain an *R*_0_ of 4.38 whereas for the SEIR model (*m =* 2, *n =* 2, 1/*σ* = 2.6 d, 1/*γ* = 2.1 d) we obtain an *R*_0_ of 16.9. Thus the initial rate of increase in incidence does well in estimating *R*_0_ for the exponentially distributed SIR model but significantly less well for the gamma-distributed SEIR model. Given that we are fitting an additional parameter, it is to be expected that a limited number of data points confounds the estimation of *R*_0_ when we include a latent period in the model assumptions. However, this also highlights that even when incorporating a latent period, estimates of *R*_0_ based on the initial epidemic growth rate may potentially underestimate the true value of *R*_0_.

### Management Consequences

**Figure 4:**
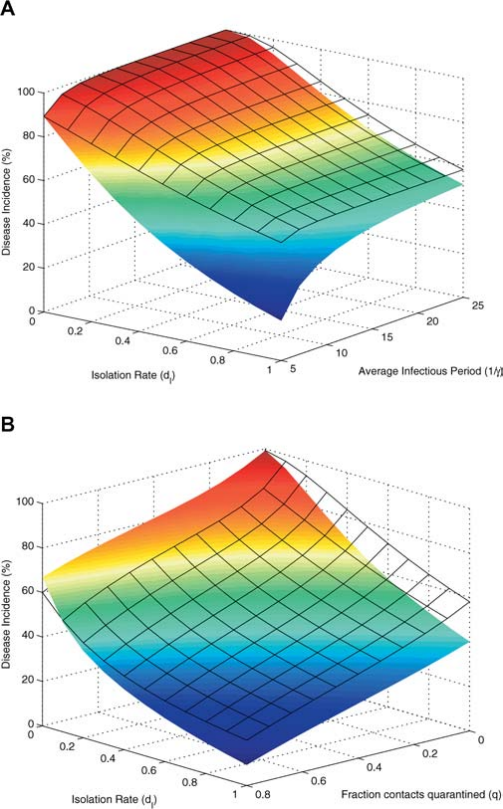
The Predicted Effectiveness of Contact Tracing and Isolation of Infected Individuals in a Population of 1 Million Susceptible Individuals (A) The proportion of the population contracting an introduced infection is depicted as a function of the infected isolation rate *(d_I_)* and the infectious period (1/*γ*). (B) The consequences of contact tracing. In both, the surfaces represent predictions of the SEIR model with an exponential (colored surface) or gamma (black grid surface; *m = n =* 10) distribution of the latent and infectious periods, respectively. Model parameters: *β =* 0.5 per day, 1/*σ* = 5 d, *τ_Q_* = 10 d, and *τ_D_* = 2 d. In (B), 1/*λ* = 10 d.

The results outlined above highlight the pitfalls of making a priori assumptions concerning the distributions of latent and infectious periods when estimating parameters. Depending on the precise details, inappropriate model selection may give rise to either gross over- or underestimates for the basic reproductive ratio of an infection. However, even when parameter estimates are reliable, choice of model structure can also be very important when making recommendations concerning individual-level control strategies. Historically, it has been shown that contact tracing and the effective quarantine of infected individuals and those potentially exposed is an important means of infection management [13,39,40]. We introduce both these measures into the SEIR epidemic model, assuming that there is a small delay in detecting newly infectious individuals, which may represent an asymptomatic phase or uncertainty in diagnosing symptoms (see Methods and Protocol S1). As we show in Figure 4, the precise levels of isolation of infected individuals and of quarantining contacts required to control the outbreak and the predicted level of disease incidence are crucially affected by whether the classic exponentially distributed SEIR model or a more realistic framework is used.

The process of isolating infected individuals results in a reduction in the mean infectious period (see Protocol S1). It is much more effective when the infectious period is exponentially distributed because it essentially truncates the tail of the distribution, so that the infectious period of a few individuals is dramatically reduced. This effect is not as pronounced in the gamma-distributed models because there is less variation in the infectious periods (see Figure 1A). In the same way, a longer delay in detecting infected individuals has fewer consequences for the exponentially distributed model because during this time many individuals will have naturally left the infectious class. Under the assumption of a gamma-distributed infectious period most individuals are infectious for a minimum period of time so early detection is more important. While the predicted difference between the exponential and gamma-distributed models depends on the duration of the infectious period and the fraction of contacts traced, it is generally true that models with an exponentially distributed infectious period will give rise to overly optimistic predictions concerning the effectiveness of isolating infected individuals.

To focus on the effects of the infectious period distribution on different courses of intervention we have assumed that all those who are quarantined and exposed are detected before the end of the quarantine period and are not released back into the general population. We have also formulated a model that takes into account the distribution of the latent period during quarantine and find similar qualitative results to those shown in Figure 4. However, if the average latent period is increased relative to the fixed quarantine period and there is only a small amount of isolation of infected individuals, then the control measures are predicted to be more effective for the gamma-distributed model, because more exposed individuals in the exponentially distributed model will leave quarantine before they develop the infection.

## Discussion

The use of models in epidemiology dates back almost a century, and while traditional models have often been highly successful in explaining observed dynamics [17,20,28,29,41], our results show that within a strict management setting, epidemiological details can make a crucial difference. Although a body of theoretical work [25,26,30] has demonstrated the importance of incorporating realistic distributions of latent and infectious periods into models of endemic disease, few studies have considered the effects associated with making predictions for an emerging disease [42].

The large discrepancies between estimates of *R*_0_ from the exponentially distributed and gamma-distributed fits reiterate the importance of accurately determining the precise distributions of latent and infectious periods. Although the data required for such a task are often available from post hoc analyses of epidemics they are certainly lacking for a novel emerging infection. Instead, the uncertainty surrounding assumptions about the distributions should be incorporated into quantitative predictions made from epidemiological models, especially since this may well be greater than any uncertainty that arises from noise in the data. Of course, more sophisticated fitting methods than those used in this paper exist [43–46], but if the underlying structure of the model is inappropriate, the method of parameterization is largely irrelevant.

The take home message from our work is that when developing models for public health use, we need to pay careful attention to the intrinsic assumptions embedded within classical frameworks. While some practitioners are already using the approach we advocate [3,15,34,39,47], the vast majority of applied epidemiological studies still use models that incorporate exponentially distributed latent and infectious periods. Perhaps this work points to the next steps in delivering quantitatively accurate epidemiological models.

## Supporting Information

Protocol S1 Further Details and Analysis of the Mathematical Models (7 KB TEX).

**Patient Summary**

**Background**

When a new infectious disease emerges, such as SARS, it is important to try to predict how the disease will behave, e.g., how infectious it is and what its latent period is, so that the spread of the disease through the population can be estimated and appropriate public health measures such as quarantining can be decided on.

**What Did the Authors Do?**

They assessed different currently used mathematical models of disease outbreaks, including models that took no account of latent periods, and another that assumed that the latent and infectious periods had a particular pattern—called exponential. They showed that both of these assumptions could potentially lead to underestimating the way the disease spreads. They then tested their predictions on a known outbreak of influenza that occurred in a school.

**What Do These Findings Mean?**

Public health officials may need to rethink the way that they try to predict outbreaks of infectious disease. Minimally, they need to be sure that they put into any model the most accurate predictions of the behavior of the disease.

**Where Can I Get More Information?**

The Health Protection Agency in the United Kingdom has a Web site that explains its work on assessing infectious disease outbreaks:

http://www.hpa.org.uk/infections/default.htm

The Centers for Disease Control and Prevention in the United States is a good place to start for information on any new infectious diseases:

http://www.cdc.gov/

## Abbreviations

SEIR: susceptible–exposed–infectious–recovered
SIR: susceptible–infectious–recovered
