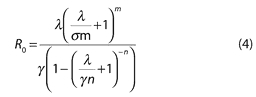# Correction: Appropriate Models for the Management of Infectious Diseases

**DOI:** 10.1371/journal.pmed.0020320

**Published:** 2005-08-30

**Authors:** Helen J Wearing, Pejman Rohani, Matt J Keeling

In *PLoS Medicine*, vol 2, issue 7, DOI: 10.1371/journal.pmed.0020174

Equation 4 was incorrectly printed. It should be as follows: